# Progression of myocardial dysfunction and prediction of arrhythmic events in patients with exercise-induced arrhythmogenic cardiomyopathy

**DOI:** 10.1016/j.hroo.2024.08.003

**Published:** 2024-08-10

**Authors:** Linda T. Aaserud, Christine Rootwelt-Norberg, Christian K. Five, Eivind W. Aabel, Nina E. Hasselberg, Erik Lyseggen, Kristina H. Haugaa, Øyvind H. Lie

**Affiliations:** 1 Department of Cardiology, Oslo University Hospital, Rikshospitalet, Oslo, Norway; 2 Institute of Clinical Medicine, Faculty of Medicine, University of Oslo, Oslo, Norway; 3 ProCardio Center for Innovation, Oslo University Hospital, Rikshospitalet, Oslo, Norway

**Keywords:** Athlete, Cardiomyopathy, Ventricular arrhythmias, Echocardiogram, Prediction

## Abstract

**Background:**

Several reports exist of an acquired exercise-induced arrhythmogenic cardiomyopathy. Little is known about myocardial disease progression and arrhythmia prediction in this population.

**Objective:**

The study sought to explore the evolution of myocardial function and structure and its relation to incident life-threatening ventricular arrhythmias (VA), to identify markers of impending events.

**Methods:**

We included athletes (individuals with exercise doses >24 metabolic equivalent of task hours per week, >6 consecutive years, participating in organized and competitive sports) who had VA, absence of family history and known genetic variants associated with cardiac disease, and no other identified etiology, in a tertiary referral single-center, longitudinal cohort study of patients with exercise-induced arrhythmogenic cardiomyopathy (EiAC). Evolution of myocardial function and structure was assessed by repeated echocardiographic examinations during long-term follow-up. Life-threatening VA were assessed at baseline and during long-term follow-up.

**Results:**

Forty-one EiAC patients (15% women, age 45 ± 13 years) were followed for 80 (interquartile range 48–115) months. There were no changes in myocardial function or structure in the overall population during follow-up. We observed high incidence rate and high recurrence rate of life-threatening VA in EiAC patients. Subtle deterioration of right ventricular function was strongly associated with subsequent first-time VA (odds ratio 1.12, 95% confidence interval 1.01–1.25, *P =* .031, per 1% deterioration of right ventricular free wall longitudinal strain).

**Conclusion:**

There were no clear changes in myocardial function or structure during follow-up in the overall population, but there was a high incidence rate and high recurrence rate of life-threatening VA. Subtle right ventricular deterioration by free wall longitudinal strain was a strong predictor of impending first-time life-threatening VA during follow-up.

## Graphical abstract

**Figure:**
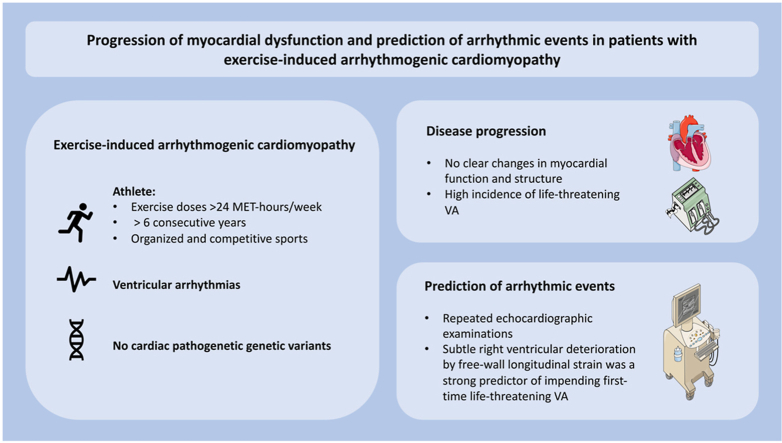

**Key Findings**

▪ There were no general detrimental changes in myocardial function or structure during long-term follow-up in the exercise-induced arrhythmogenic cardiomyopathy population.
▪ We observed high incidence rate and high recurrence rate of life-threatening ventricular arrhythmias.
▪ Subtle right ventricular deterioration by free wall longitudinal strain was a strong predictor of impending first-time life-threatening ventricular arrhythmia during follow-up.

## Introduction

The benefits of regular physical activity are well established. However, growing evidence supports that sustained and vigorous endurance exercise may increase the risk of cardiac arrhythmias in susceptible individuals.^1^

Cardiomyopathies are reported to be the leading cause of exercise-related sudden cardiac death in young people in our parts of the world.^2^^,^^3^ Exercise may even promote the disease progression of some of the inheritable cardiomyopathies.^4^, ^5^, ^6^, ^7^ Furthermore, a controlled study has reported about the effect of exercise when desmosomal vulnerability is present.^8^

Several reports also exist of an acquired exercise-induced clinical entity.^1^^,^^9^, ^10^, ^11^, ^12^, ^13^ The understanding and nomenclature have evolved over time, and there is no clear consensus on recognition of the entity; consequently, these reports use slightly different definitions and describe somewhat different populations. While the most notable characteristic initially was the resemblance to inheritable arrhythmogenic right ventricular cardiomyopathy (ARVC), the clinical condition is now associated with both abnormalities involving the right ventricle (RV), the left ventricle (LV), and the right ventricular outflow tract (RVOT). The proposed term exercise-induced arrhythmogenic cardiomyopathy (EiAC) reflects this clinical syndrome of various cardiac injuries and serious ventricular arrhythmias (VA) observed in athletes.^14^, ^15^, ^16^

Previous studies are few and small and not suited for conclusive inference about disease progression and arrhythmia prediction in this population.

We aimed to explore the evolution of myocardial function and structure during long-term follow-up in patients with EiAC, and its relation to incident life-threatening VA to identify markers of impending events.

## Methods

### Study design and population

We evaluated subjects from a previous report,^13^ referred for evaluation and therapeutic management at the Department of Cardiology, Oslo University Hospital, Rikshospitalet, between 2004 and 2017 for inclusion in a tertiary referral single-center longitudinal cohort study.

To ensure a comprehensive consideration of the diagnosis and to capture its various clinical expressions, we included athletes (defined as individuals with present or previous exercise doses >24 metabolic equivalent of task (MET)-hours per week for >6 consecutive years, participating in organized and competitive sports) who had VA (premature ventricular contractions, ventricular tachycardia, and ventricular fibrillation [VF]), given absence of family history or known genetic variants associated with cardiac disease, and no other identified etiology. Consequently, athletes with a phenotype meeting the 2010 Task Force Criteria (TFC) for ARVC diagnosis were included in our cohort in the absence of heredity.^17^

Exercise habits were assessed using a standardized, nonvalidated interview form (Supplemental Table 1). All were recommended to avoid high-intensity and competitive exercise once the diagnosis was established, but individual exercise dose was not obtained during follow-up.

We assessed family history of premature cardiac disease and sudden cardiac death and performed genotyping by next-generation sequencing analysis to exclude genetic variants associated with inheritable ARVC and other relevant cardiomyopathies and channelopathies when appropriate.

Patients underwent comprehensive diagnostic workup to exclude other known etiologies of VA. Invasive coronary angiography was performed on clinical indication to exclude coronary artery disease. Patients <35 years of age without coronary artery disease suspicion underwent noninvasive tests (computed tomography angiography or bicycle stress electrocardiogram [ECG]). Cardiac magnetic resonance imaging or positron emission tomography scan was performed to exclude inflammatory etiology. Patients with echocardiographic signs of shunts and moderate-to-severe valvular disease were excluded.

All patients provided written informed consent for inclusion in a registry. The study was approved by the Regional Committee for Medical and Health Research Ethics of South-Eastern Norway, in accordance with the Declaration of Helsinki.

### Outcome

Life-threatening VA was defined as syncope (sudden loss of consciousness without warning symptoms, rapid awakening to full consciousness) with simultaneous documented nonsustained ventricular tachycardia (VT) (≥3 consecutive ventricular contractions with rate >100 beats/min), sustained VT (> 30 seconds), appropriate therapy from an implantable cardioverter-defibrillator (ICD), or aborted cardiac arrest (ACA).^18^ Systematic adjudication of outcome was performed by a clinical cardiologist blinded to exposure data. Adjudication by consensus between clinical cardiologists was performed in cases of ambiguous medical record reporting.

Presence of life-threatening VA was assessed at baseline and during follow-up. In patients without documented life-threatening VA at baseline, presence of documented VA during follow-up was defined as first-time life-threatening VA. In patients with already documented life-threatening VA at baseline, documented VA during follow-up was defined as recurrent life-threatening VA.

### Electrocardiography

Repeated 12-lead ECGs obtained from outpatient and referring clinics were assessed according to international recommendations and the 2010 TFC for major repolarization and depolarization abnormalities. Reports from in-hospital arrhythmia monitoring, Holter monitoring, and ICD transcripts were assessed for life-threatening VA.

### Echocardiography

Repeated comprehensive echocardiographic examinations (Vivid 7, E9, or E95; GE Vingmed Ultrasound) were performed at baseline and during long-term follow-up in the outpatient clinic. Key parameters of cardiac function and structure were assessed offline (EchoPac version 202; GE Vingmed Ultrasound) by 1 single expert observer blinded to outcome data.^19^ LV function was assessed by LV ejection fraction calculated by Simpson’s biplane method and global longitudinal strain from a 16-segment LV model. RV function was assessed by fractional area change, tricuspid annular plane systolic excursion, and right ventricular free wall longitudinal strain (RVFWSL) from a 3-segment RV model. LV dimensions included LV end-diastolic diameter and RV dimensions included proximal right ventricular outflow tract (RVOT) diameter assessed in the parasternal short-axis view and basal right ventricular diameter.^20^

### Statistical analyses

Values are presented as mean ± SD, median (interquartile range), or frequency and percentage and compared by Student’s *t* test, Fisher’s exact test, or the Mann-Whitney *U* test, as appropriate. Statistical testing was not performed in cases of very low rates of observations. Progression of myocardial structure and function during long-term follow-up was assessed by linear mixed model regression using random intercept and random slope, and unstructured covariance structure. Progression analyses were performed on the overall population and repeated within the subgroups with and without life-threatening ventricular arrhythmias by last follow-up, and within the subgroup of patients with first-time life-threatening VA during follow-up. The trajectories of parameters of myocardial structure and function were visualized using fit plots with 95% confidence intervals for the subgroups with and without life-threatening VA by last follow-up. We used univariate Cox regression analyzes to explore possible risk factors for first-time life-threatening VA. The impact of generalized disease progression on risk of first-time life-threatening VA was assessed by systematic exploratory generalized estimation equations using random effects at individual patient level and unstructured covariance structure. Kaplan-Meier analysis was used to study survival free from life-threatening VA during follow-up, with log-rank testing to compare across the subgroups with and without life-threatening VA at baseline. Two-sided *P* values <.05 were considered significant. All analyzes were performed in Stata SE 17.0 (StataCorp).

## Results

### Study population

Of 43 previously reported EiAC patients, we included 41 patients (15% women, age 45 ± 13 years at baseline). Of the 2 excluded patients, 1 had a positive genotype for inheritable ARVC in an extended repeated genetic test performed on clinical indication and 1 had developed progressive coronary artery disease and aortic valve stenosis. The population had high average exercise dose and accumulated exercise duration (Table 1). Seventeen of the athletes were engaged in combined sport activities, 7 were runners, 5 did team sports (football, ice hockey and basketball), 5 were cyclists, 4 were skiers, 2 were swimmers, and 1 was a rower. Median follow-up time was 80 (interquartile range 48–115) months. When applying the 2010 TFC, 34% of the patients had a definite diagnosis, 12% had a borderline diagnosis, 34% had a possible diagnosis, and 20% did not meet the criteria (Supplemental Table 2).

**Table 1:** Baseline characteristics and echocardiographic key parameters

	EiAC total (N = 41)	EiAC non-LTVA (n = 30)	EiAC LTVA (n = 11)	*P*
Characteristics				
Age, y	45 ± 13	46 ± 13	44 ± 14	.79
BMI, kg/m^2^	25 ± 3	26 ± 3	23 ± 3	.03
BSA, m^2^	2.1 ± 0.2	2.1 ± 0.2	1.99 ± 0.2	.12
Height, cm	183 ± 8	184 ± 8	183 ± 9	.81
Weight, kg	85 ± 14	88 ± 13	79 ± 14	.07
SBP, mm Hg	135 ± 18	138 ± 16	125 ± 20	.08
DBP, mm Hg	82 ± 12	82 ± 12	80 ± 13	.64
Exercise dose, MET-hours	9893 (5200–20,000)	10,088 (7019–20,389)	7215 (3120–18,200)	.21
MET-hours/year	3686 (2080–5294)	3907 (2219–6230)	3029 (2080–5250)	.78
Hours per year	388 (260–600)	441 (251–671)	366 (260–600)	.97
Intensity, METs	9 (8.5–10)	9 (9–10)	8.5 (7.5–9)	.03
Follow-up, mo	80 (48–115)	70 (42–106)	112 (91–178)	<.001
Medication				
Beta-blocker	27	17 (56)	10 (90)	.06
Calcium-channel blocker	2	2 (7)	0	
Amiodarone	2	0	2 (18)	
Flecainide	3	3 (10)	0	
Echocardiography				
LVEF, %	55 ± 4	55 ± 4	56 ± 6	.46
GLS, %	–15.2 ± 2.7	–15.2 ± 2.4	–15.2 ± 3.8	.98
TAPSE, mm	25 ± 5	26 ± 5	24 ± 4	.15
FAC, %	39 ± 8	38 ± 8	42 ± 8	.15
RVFWSL, %	–23.1 ± 4.5	–24.4 ± 3.6	–19.7 ± 4.9	.01
RVOT, mm	39 ± 6	40 ± 6	36 ± 7	.13
RVD, mm	48 ± 7	49 ± 6	47 ± 9	.43

Characteristics and echocardiographic key parameters in 41 EiAC patients, comparing patients without and with life-threatening VA at baseline. Values are mean ± SD, median (interquartile range), or frequencies with percentages, and compared by unpaired Student’s *t* test, Mann-Whitney *U* test, or Fisher’s exact test as appropriate.

BMI = body mass index; BSA = body surface area; DBP = diastolic blood pressure; EiAC = exercise-induced arrhythmogenic cardiomyopathy; FAC = fractional area change; GLS = global longitudinal strain; LVEF = left ventricular ejection fraction; LTVA = life-threatening ventricular arrhythmia; MET = metabolic equivalent of task; RVD = right ventricular diameter; RVFWSL = right ventricular free wall longitudinal strain; RVOT = right ventricular outflow tract; SBP = systolic blood pressure; TAPSE = tricuspid annular plane systolic excursion; VA = ventricular arrhythmias.

### Arrhythmic outcome

Life-threatening VA had occurred in 11 (27%) athletes at baseline, of which 5 presented as ACA, 5 were diagnosed with sustained VT, and 1 had a documented syncopal nonsustained VT. Recurrent life-threatening VA during follow-up were registered in 5 (45%) of these patients, of which 4 presented with appropriate ICD shocks and 1 had sustained VT without shock therapy due to inappropriate ICD programming (Supplemental Table 3).

Six (20%) of 30 athletes without previous events experienced first-time life-threatening VA during follow-up, of which 1 presented with ACA, 2 experienced sustained VT, and 3 received appropriate therapy from a primary prevention ICD (Supplemental Table 3).

**Figure 1:**
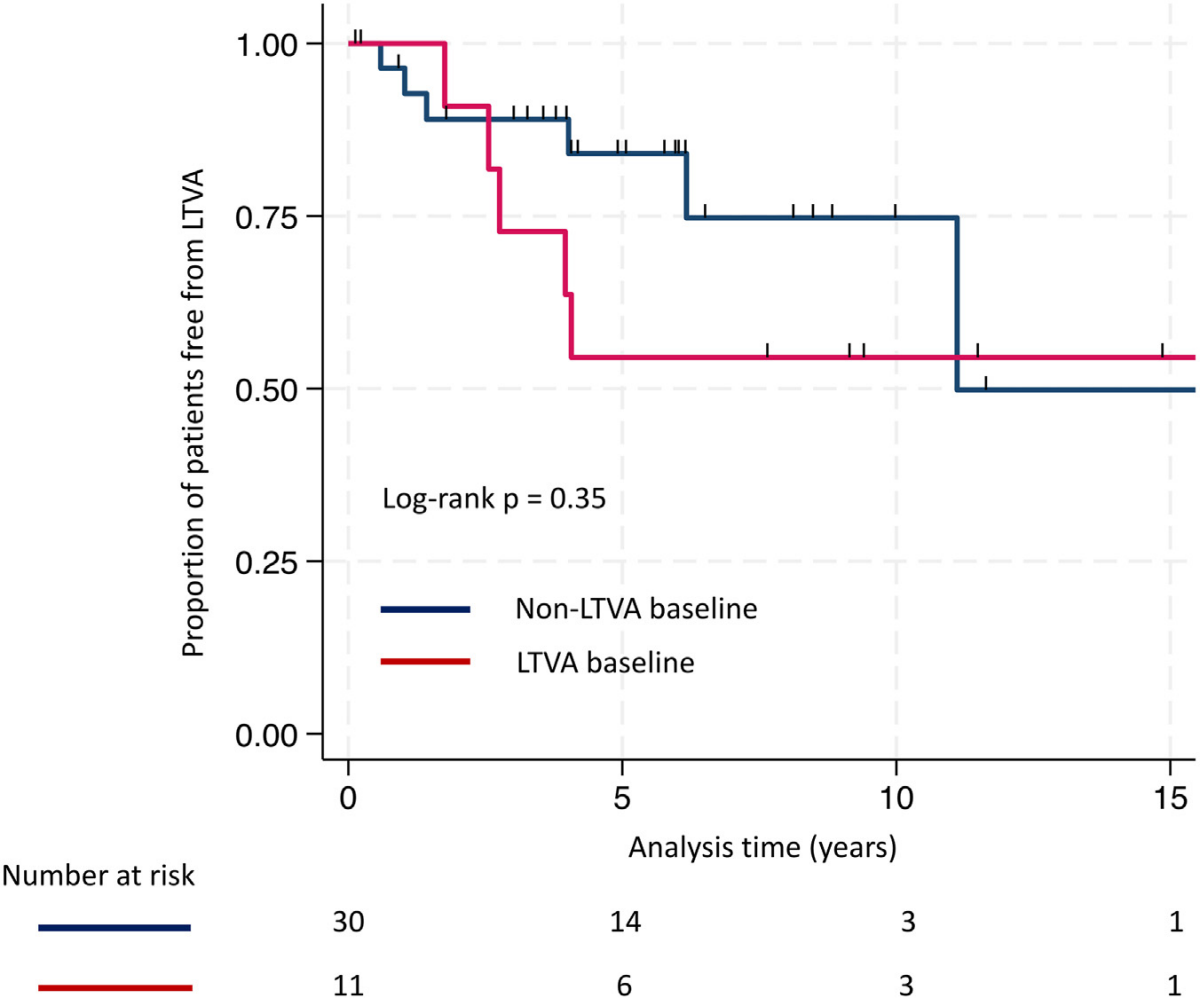
Survival free from life-threatening ventricular arrhythmias (LTVA). Kaplan-Meier analysis demonstrated related survival free from LTVA between the exercise-induced arrhythmogenic cardiomyopathy subgroups without (blue line) and with (red line) LTVA at baseline.

In total, 17 (41%) of the athletes in our cohort had experienced life-threatening VA by last follow-up. The risk of experiencing life-threatening VA during follow-up did not clearly differ between patients with or without previously documented life-threatening VA at baseline (hazard ratio 1.75, 95% confidence interval [CI] 0.53 to 5.79, *P =* .36, for history of life-threatening VA) (Figure 1).

### Electrocardiography

There was a low prevalence of ECG abnormalities according to the 2010 TFC for major repolarization and depolarization. No patients had Epsilon waves at baseline or during follow-up. T-wave inversions in V1 to V3 were registered in 6 patients at baseline and in additional 4 patients during follow-up. ECG abnormalities were observed both in the subgroup with and without life-threatening VA (8 patients with VA, 2 patients without VA). The low prevalence did not allow for statistical testing.

### Echocardiographic disease progression

Baseline echocardiographic parameters of RV and LV function were normal to subnormal, while RV dimensions were increased (Table 1). There was no difference between the patients with or without previous life-threatening VA, except from a worse RV function by RVFWSL in those having experienced life-threatening VA.

**Figure 2:**
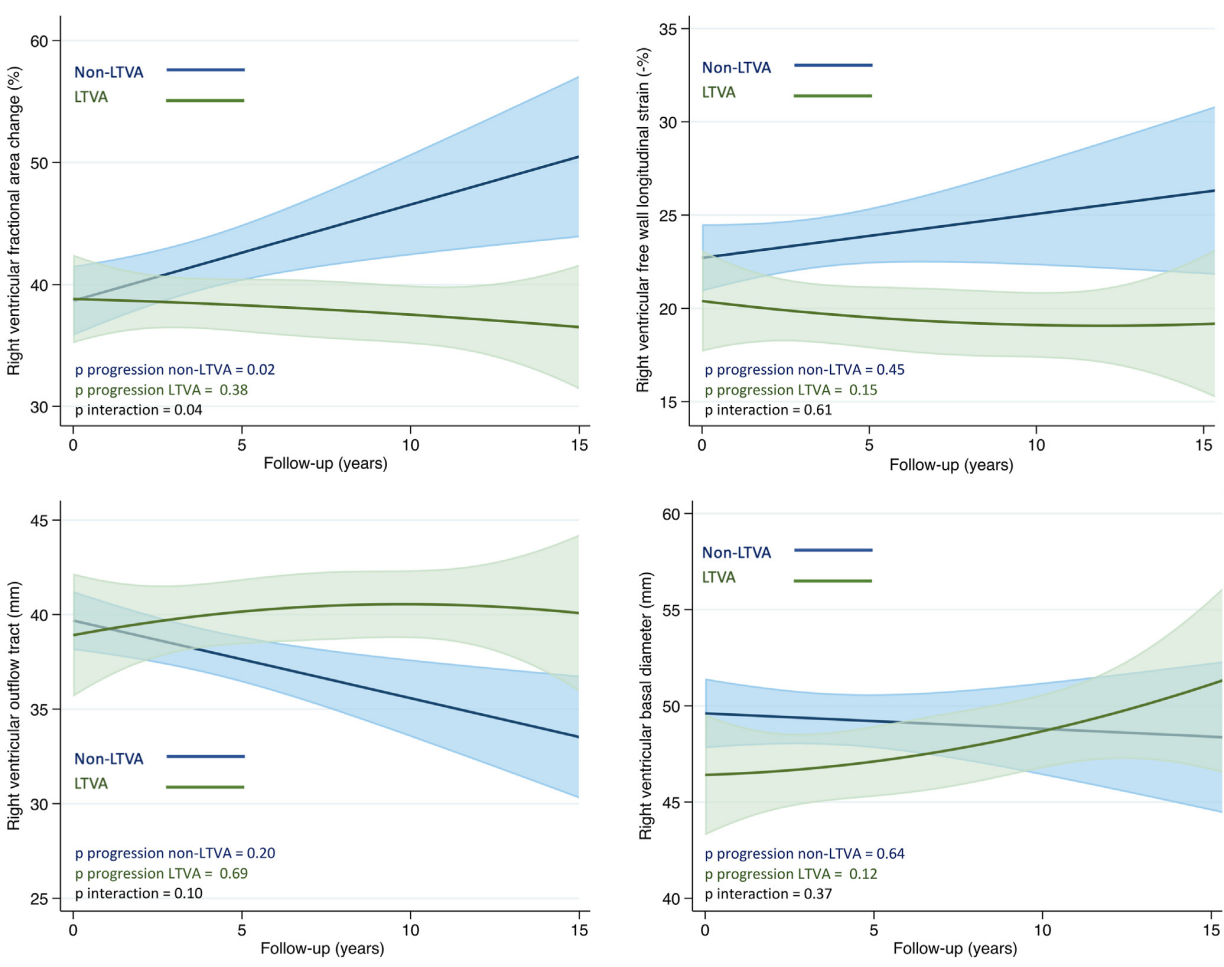
Right ventricular myocardial function and structure during follow-up. There were no changes in right ventricular myocardial function or structure during follow-up, comparing the exercise-induced arrhythmogenic cardiomyopathy subgroups with (green fit plot with corresponding 95% confidence interval) and without (blue fit plot with corresponding 95% confidence interval) life-threatening ventricular arrhythmias (LTVA) by last follow-up.

During follow-up, there were no changes in myocardial function or structure in the overall population (Table 2) or in the 17 patients who had experienced life-threatening VA by last follow-up (Figure 2, Supplemental Table 4a). However, when evaluating only patients with first-time life-threatening VA during follow-up, we observed signs of RV deterioration by RVFWSL (yearly progression rate 0.45, 95% CI 0.01 to 0.88, *P =* .04) (Supplemental Table 4b). Patients with a less arrhythmogenic phenotype had signs of normalization of RV function with an increase in RV fractional area change during follow-up (yearly progression rate 0.55, 95% CI –0.08 to 1.03, *P =* .02) (Figure 2, Supplemental Table 4a).

**Table 2:** Yearly progression rates of echocardiographic key parameters

	Progression rate at 1 y (SE)	95% CI	*P*
LVEF	–0.05 (0.08)	–0.21 to 0.11	.54
GLS	–0.04 (0.05)	–0.13 to 0.06	.45
TAPSE	–0.05 (0.16)	–0.26 to 0.36	.76
FAC	0.02 (0.15)	–0.28 to 0.33	.86
RVFWSL	0.17 (0.11)	–0.04 to 0.38	.11
RVOT	0.02 (0.13)	–0.24 to 0.27	.89
RVD	0.14 (0.10)	–0.05 to 0.34	.15

There was no deterioration of myocardial function or dimensions during follow-up in the EiAC population.

CI = confidence interval; EiAC = exercise-induced arrhythmogenic cardiomyopathy; FAC = fractional area change; GLS = global longitudinal strain; LVEF = left ventricular ejection fraction; RVD = right ventricular diameter; RVFWSL = right ventricular free wall longitudinal strain; RVOT = right ventricular outflow tract; TAPSE = tricuspid annular plane systolic excursion.

### Echocardiographic predictors of first-time VA

Deterioration of RV function by RVFWSL was strongly associated with subsequent first-time life-threatening VA. The odds of an impending first-time life-threatening VA during follow-up increased with deterioration of RV strain values (odds ratio 1.12, 95% CI 1.01 to 1.25, *P =* .03, per 1% worsening RVFWSL from the previous echocardiographic exam). Other RV function parameters, increasing RV dimension, or deterioration of LV function were not predictors of first-time life-threatening VA (Table 3).

**Table 3:** Echocardiographic predictors of first-time life-threatening VA

	Odds ratio	95% CI	*P*
LVEF	0.96	0.86–1.1	.48
GLS	1.1	0.88–1.33	.47
TAPSE	0.94	0.82–1.1	.31
FAC	0.96	0.89–1.02	.19
RVFWSL	1.12	1.01–1.25	.03
RVOT	1.05	0.93–1.19	.41
RVD	0.96	0.87–1.07	.46

The odds of an impending first-time life-threatening VA during follow-up increased with deterioration of right ventricular strain values. Other echocardiographic key parameters were not predictors of first-time life-threatening VA.

CI = confidence interval; FAC = fractional area change; GLS = global longitudinal strain; LVEF = left ventricular ejection fraction; TAPSE = tricuspid annular plane systolic excursion; RVD = right ventricular diameter; RVFWSL = right ventricular free wall longitudinal strain; RVOT = right ventricular outflow tract; VA = ventricular arrhythmia.

## Discussion

The present study describes long-term follow-up in a cohort of patients diagnosed with EiAC. Despite no general detrimental changes in myocardial function or structure, we observed high incidence rate and high recurrence rate of life-threatening VA, emphasizing EiAC as a highly arrhythmogenic condition. Risk stratification is challenging in these individuals, but our observations suggest that follow-up with repeated echocardiographic examinations may have important value to detect subtle functional disease progression indicating increased arrhythmic risk.

### Myocardial structure and function

The EiAC patients in this study presented a wide spectrum of structural and functional abnormalities, and illustrate the heterogeneity of clinical phenotypes enclosed by the proposed EiAC diagnosis.

The baseline observations demonstrated the considerable overlap with both the athlete’s heart and inheritable ARVC. Findings of normal to subnormal RV and LV function, and increased RV dimensions are related to the normal physiological cardiac adaptation to increased workload known as the athlete’s heart. The right-sided cardiac structures are more vulnerable to the increased workload during athletic exercise, and right ventricular enlargement is reported in healthy athletes.^21^ Conversely, inheritable ARVC predominantly affects the right side of the heart, and these features may also represent early manifestations of inheritable ARVC.^22^ Furthermore, a significant proportion of the patients even had a definite or borderline ARVC diagnosis when applying the 2010 TCF criteria. A clear differentiation between EiAC and inheritable ARVC may also be challenging, and possibly even unattainable, due to a large possible spectrum of genetic vulnerability. Both non–exercise-induced and exercise-induced conditions likely involve unidentified genetic factors.

Notably, we observed no general detrimental changes in myocardial structure or function during long-term follow-up in the overall EiAC population. This evolution was not typical of either the athlete’s heart or inherited ARVC. The EiAC patients were given exercise restrictions once diagnosed, but subsequent individual exercise doses and compliance with these restrictions were not obtained during follow-up. It is possible that the athletes restricted their exercise, potentially accounting for the absence of change, thus suggesting a beneficial response to exercise restriction. Conversely, it is also possible that they did not adhere to the exercise restrictions, and that exercise may not have been the etiological factor for the cardiomyopathy, or that restricting exercise provided no observable benefits.

A significant overlap with both the athlete’s heart and inheritable ARVC presents one of the main challenges in this diagnosis. Differentiation of EiAC is difficult based on the initial evaluation alone, while repeated echocardiographic examinations and repeated exercise assessment during follow-up can provide important information about adaptation to detraining or disease progression. Further studies are needed to enhance our understanding of the differences between physiological and pathological remodeling in the heart of athletes.

### Prediction of life-threatening VA

The high incidence and recurrence rate of life-threatening VA in the athletes followed at our center are in line with other corresponding reports and underline EiAC as a highly arrhythmogenic condition.^10^

Risk stratification in these individuals is challenging, and the initial evaluation and management of EiAC patients in our cohort did not sufficiently inform risk prediction. However, previous reports have described patchy myocardial fibrosis in athletes, both from murine models and cardiac magnetic resonance imaging, while isolated subepicardial RVOT scars have been reported in endurance athletes with ventricular arrhythmias.^11^^,^^15^^,^^23^ A previous study even found positive programmed ventricular stimulation to be strongly associated with ventricular arrhythmic events during follow-up of endurance athletes, and indicate that electrophysiological studies may have value in risk stratification of EiAC patients.^10^

Nevertheless, all our patients underwent repeated echocardiographic examinations at baseline and during long-term follow-up. We observed that deterioration of RV function, as measured by RVFWSL, seems to be a strong predictor of impending life-threatening VA and could be valuable in the risk prediction of these patients. The odds of an impending first-time life-threatening VA in a given patient increased by 12% with every 1% deterioration in RVFWSL from one exam to the next. One percent change in RVFWSL is likely to be within measurement error even in inexperienced users, but the present results also suggest that a 5% absolute deterioration may increase the odds by 60%. This finding is also supported by a more deteriorated RVFWSL in patients with present life-threatening VA at baseline and subtle worsening RV function in those who experienced first-time life-threatening VA during follow-up, indicating that RV disease progression is associated with increased arrhythmic risk.

There is a need for repeated risk stratification during close follow-up and improved tools for clinical decision making for EiAC patients. The proarrhythmic substrate as well as the role of electrophysiological studies implicated in EiAC should be assessed in future studies to improve risk prediction in the EiAC population. For the moment, our observations indicate that subtle myocardial dysfunction, as detected by widely available echocardiographic strain, is of prognostic value. Following individuals with repeated examinations, despite only subtle changes within normal reference values, is important for detecting functional disease progression that indicates increased arrhythmic risk.

### Limitations

This was a tertiary referral single-center, longitudinal cohort study with inherent limitations. The cohort is small and highly selected, and the observations are subject to selection bias and survival bias. The cohort consists of predominantly male athletes, but this is in line with other comparable studies. EiAC patients who were considered to have very low risk were transferred back to primary centers, while the remaining patients were followed over a longer period. Overestimation of the true arrhythmic incidence is therefore likely. Exercise history and doses were self-reported inducing reporting bias and recall bias. No quantification of exercise was performed during follow-up, and speculations and hypothesis of a noncompliant exercise behavior cannot be formally tested. Idiopathic VF is a recognized clinical entity and may also be covered by our inclusion criteria. However, the temporal association of the deterioration of the right ventricle preceding incident VA during follow-up is unlikely to be biased by possible presence of idiopathic VF. It is possible, even likely, that yet unidentified genetic variants, or those not considered during genotyping, could influence the EiAC phenotype and increase the susceptibility of certain athletes to arrhythmias.

### Conclusions

There were no clear changes in myocardial function and structure during follow-up in the overall EiAC population, but there were a high incidence rate and a high recurrence rate of life-threatening VA. Subtle worsening of RV function was observed in those who experienced first-time life-threatening VA during follow-up, and deterioration of RV function was a strong predictor of impending first-time life-threatening VA. These findings may inform clinical decision making and management of EiAC patients.
