# Supplementary material for: Progression of myocardial dysfunction and prediction of arrhythmic events in patients with exercise-induced arrhythmogenic cardiomyopathy
Source: Heart Rhythm O2. 2024 Aug 10;5(10):705–12. doi: 10.1016/j.hroo.2024.08.003 (PMC11549643; doi:10.1016/j.hroo.2024.08.003)
Supplement: Supplemental Tables S1–S4 [file mmc1.docx]

## **SUPPLEMENTARY MATERIAL**

## **Supplementary Table 1**

**Questionnaire - Exercise history starting at school age**

Physical activity in the purpose of exercise training

- One or several activities
- Exercise level:
  - 1. Low-intensity (forehead sweating)
  - 2. Moderate-intensity (exercise, shortness of breath)
  - 3. High-intensity (competitive intensity)

Changes in intensity must be reported

- Exercise hours during a week
- Number of months during a year
- No exercise training is to be registered

| **Type of activity** | **Level/intensity** | **Age (exercise debut)** | **Age (end of exercise)** | **Hours per week** | **Months per year** | **Notes** |
| --- | --- | --- | --- | --- | --- | --- |
| Swimming | 2 | 8 years | 15 years | 2 | 9 | Free during the summer holiday |
|  |  |  |  |  |  |  |
|  |  |  |  |  |  |  |
|  |  |  |  |  |  |  |

**Supplementary Table 2** – Distribution of diagnostic categories according to the 2010 Task Force Criteria

| **Diagnostic categories** |  | **Major and minor criteria** |
| --- | --- | --- |
| Global or regional dysfunction or structural abnormalities determined by echocardiography, magnetic resonance imaging, or right ventricular angiography |  | Major: 15  Minor: 6 |
| Tissue characterization of wall |  | Major: 0  Minor: 0 |
| Repolarization abnormalities |  | Major: 9  Minor: 1 |
| Depolarization abnormalities |  | Major: 1 Minor: 26 |
| Arrhythmias |  | Major: 7  Minor: 22 |
| Family history |  | Major: 0  Minor: 0 |
| **Diagnosis** |  | Definite: 14 (34%)  Borderline: 5 (12%)  Possible: 14 (34%)  Non-criteria: 8 (20%) |

The 2010 Task Force Criteria for ARVC with major and minor criteria and diagnostic categories for the EiAC population.

**Supplementary Table 3** - Patients registered with life-threatening VA during follow-up (recurrent and first-time).

| **Referral condition** | **Initial diagnosis** | **Management** | **Time to event (years)** | **LTVA during follow-up** | **Situation** |
| --- | --- | --- | --- | --- | --- |
| **Recurrent LTVA** |  |  |  |  |  |
| Syncopal NSVT | Athlete’s heart, mutation negative ARVC | BB  ICD | 2.56 | VT >200, ICD-therapy (ATP x 6 and shock x 1, all on the same day) | No trigger |
| ACA | RVOT VT, myocarditis sequelae | BB  SP ICD | 1.76 | VT 170-180, Amiodarone, “inappropriate ICD programming” | Cabin trip, alcohol |
| ACA | Borderline ARVC, EiAC | BB  SP ICD | 3.96 | ICD therapy (shock x 3) | CrossFit |
| VT | Mutation negative ARVC | BB  SP ICD | 2.76 | ICD therapy (shock) | Stress, dehydration, coffee and alcohol |
| VT | Mutation negative ARVC | BB  SP ICD | 4.07 | ICD therapy (shock) | At home |
| **First-time LTVA** |  |  |  |  |  |
| Cardiac syncope during physical activity | EiAC  Cardiac stress test: frequent PVCs and NSVT | BB  Exercise restrictions | 1.03 | VT 190 | Bike ride, awake |
| Heart palpitations, NSVT | Mutation negative ARVC or athlete´s heart | Flecainide and BB  Exercise restrictions  RFA RVOT tachycardia | 11.11 | VT 150-160 | Discontinuation of medications before RFA consideration |
| Heart palpitations and presyncopes during exercise | Mutation negative cardiomyopathy, unspecified | BB  Positive VT stim study: PP ICD  Recommended moderate-intensity exercise | 0.59 | ICD therapy (ATP x 7 and shock x 1, all on the same day) | Hiking and alcohol |
| Heart palpitations | Athlete’s heart or genetic negative cardiomyopathy, unspecified | BB  No initial exercise restrictions | 4.02 | ACA | Found next to his bicycle |
| PVCs and NSVT | RVOT VT | BB  RFA  Positive VT stim study: PP ICD | 1.43 | ICD therapy  (ATP x 3, all on the same day) | After an intensive exercise session |
| Syncopes and heart palpitations during exercise | Idiopathic VT | BB  No initial exercise restrictions  Positive VT stim study: PP ICD | 6.17 | ICD therapy  (ATP x 1 + shock x 3, all on the same day) | During sleep |

ACA = aborted cardiac arrest; AF = atrial fibrillation; AFlu = atrial flutter; ARVC = arrhythmogenic right ventricular cardiomyopathy; ATP = Antitachycardia pacing; BB = betablocker; EiAC = exercise-induced arrhythmogenic cardiomyopathy; ICD = implantable cardioverter device; LTVA = life-threatening ventricular arrhythmia; NSVT = non-sustained ventricular tachycardia; PP = Primary preventive; PVC = Premature ventricular contractions; RFA = radiofrequency ablation; RVOT = right ventricular outflow tract; SP = Secondary preventive; Stim = stimulation; SVT = supraventricular arrhythmia; VA = ventricular arrhythmia; VT = Ventricular tachycardia.

**Supplementary Table 4** - Yearly progression rate of echocardiographic key parameters within the subgroups.

**4a**

|  | **Progression rate**  **1 year (SE)** | **95 % CI** | **p** | **Progression rate**  **1 year (SE)** | **95% CI** | **p** |
| --- | --- | --- | --- | --- | --- | --- |
|  | **LTVA**  **n = 17** |  |  | **Non-LTVA**  **n = 24** |  |  |
| **FAC, %** | -0.16 (0.18) | -0.51 – 0.19 | 0.38 | 0.55 (0.24) | -0.08 – 1.03 | 0.02 |
| **RVFWSL, %** | 0.16 (0.11) | -0.06 – 0.37 | 0.15 | 0.18 (0.24) | -0.29 – 0.64 | 0.45 |
| **TAPSE, mm** | 0.03 (0.14) | -0.25 – 0.31 | 0.85 | 0.11 (0.27) | -0.43 – 0.65 | 0.69 |
| **RVOT, mm** | 0.09 (0.21) | -0.33 – 0.50 | 0.69 | -0.13 (0.10) | -0.33 – 0.07 | 0.20 |
| **RVD, mm** | 0.23 (0.15) | -0.06 – 0.52 | 0.12 | 0.06 (0.12) | -0.18 – 0.29 | 0.64 |
| **LVEF, %** | -0.06 (0.12) | -0.29 – 0.17 | 0.60 | 0.07 (0.16) | -0.24 – 0.37 | 0.67 |
| **GLS, %** | -0.10 (0.06) | -0.22 – 0.03 | 0.12 | -0.03 (0.06) | -0.14 – 0.09 | 0.62 |

**4b**

|  | **Progression rate**  **1 year (SE)** | **95 % CI** | **p** |
| --- | --- | --- | --- |
|  | **First-time LTVA FU**  **n = 6** |  |  |
| **FAC, %** | 0.03 (0.51) | -0.97 – 1.04 | 0.95 |
| **RVFWSL, %** | 0.45 (0.22) | 0.01 – 0.88 | 0.04 |
| **TAPSE, mm** | 0.181 (0.32) | -0.44 – 0.80 | 0.57 |
| **RVOT, mm** | -0.38 (0.40) | -1.17 – 0.41 | 0.35 |
| **RVD, mm** | 0.25 (0.20) | -0.15 – 0.65 | 0.22 |
| **LVEF, %** | 0.16 (0.21) | -0.26 – 0.57 | 0.46 |
| **GLS, %** | -0.21 (0.11) | -0.43 – 0.01 | 0.05 |

There was no deterioration of myocardial function or structure during follow-up in EiAC patients with or without life-threatening VA by last follow-up, but the subgroup of patients with first-time life-threatening VA during follow-up had RV deterioration by RVFWSL.

CI = confidence interval; EiAC = exercise-induced arrhythmogenic cardiomyopathy; FAC = fractional area change; FU = follow-up; GLS = global longitudinal strain; LVEF = left ventricular ejection fraction; TAPSE = tricuspid annular plane systolic excursion; RVD = right ventricular diameter; RVFWSL = right ventricular free wall longitudinal strain; RVOT = right ventricular outflow tract; SE = standard error; VA = ventricular arrhythmias.
